# Do honeybees (*Apis mellifera*) differentiate between different pollen types?

**DOI:** 10.1371/journal.pone.0205821

**Published:** 2018-11-07

**Authors:** Fabian A. Ruedenauer, Christine Wöhrle, Johannes Spaethe, Sara D. Leonhardt

**Affiliations:** 1 Department of Animal Ecology and Tropical Biology, Biozentrum, University of Würzburg, Würzburg, Germany; 2 Department of Behavioral Physiology and Sociobiology, Biozentrum, University of Würzburg, Würzburg, Germany; University of North Carolina at Greensboro, UNITED STATES

## Abstract

Bees receive nectar and pollen as reward for pollinating plants. Pollen of different plant species varies widely in nutritional composition. In order to select pollen of appropriate nutritional quality, bees would benefit if they could distinguish different pollen types. Whether they rely on visual, olfactory and/or chemotactile cues to distinguish between different pollen types, has however been little studied. In this study, we examined whether and how *Apis mellifera* workers differentiate between almond and apple pollen. We used differential proboscis extension response conditioning with olfactory and chemotactile stimulation, in light and darkness, and in summer and winter bees. We found that honeybees were only able to differentiate between different pollen types, when they could use both chemotactile and olfactory cues. Visual cues further improved learning performance. Summer bees learned faster than winter bees. Our results thus highlight the importance of multisensory information for pollen discrimination.

## Introduction

Social bees collect nectar and pollen from flowers to nourish their colony and simultaneously transfer pollen between flowers, which is crucial for the reproduction and conservation of about 80% of all flowering plant species worldwide [1, 2]. The nutrient content of pollen differs strongly between different plant species [3] and is directly linked to bee health [4–7]. In fact, imbalanced diets may play a significant role in the observed decline of honeybee colonies [8], because the nutritional state of a colony strongly affects its health and fitness [5, 9, 10]. Consequently, a nutritionally balanced diet strengthens the entire colony, and well-nourished honeybees are generally more resistant to pathogen infections or other stressors [4, 9–11]. While, for honeybees, nutritional requirements have been well defined (e.g. [5, 6, 10, 12, 13]), sensory modalities involved in resource selection based on nutritional criteria have received less attention, which is particularly true for pollen foraging [14]. While nectar provides predominantly carbohydrates, pollen supplies both larvae and adult bees additionally with essential macro- and micro-nutrients [3], including proteins [13], lipids [15–17], inorganic compounds [18] and vitamins [19]. Pollen consequently represents a very complex mixture of different chemical substance classes (also including non-nutritional plant secondary metabolites). It is typically collected from a large spectrum of different plant species [20], with pollen nutrient content strongly differing between different species [3]. Colonies would therefore benefit if foragers assessed pollen nutritional composition (henceforth referred to as pollen quality) at flowers and distinguished between different pollen types differing in nutrient content [4, 21, 22]. However, the chemical complexity of pollen renders this task challenging, as it confronts bees with a large variety of different chemical cues, which could (in theory) be used to infer quality and thus for differentiation. Pollen foragers are further likely influenced by additional (non-nutrient related) factors, such as the current provisional state of the colony [23], weather conditions or season [24, 25], which may affect their nutritional target and thus choice of chemical cue used for differentiation. Although most nutrients are inaccessibly stored within the pollen cell walls, some nutrients, such as amino acids and lipids, can easily be accessed without digestion [26] and may therefore represent promising cue candidates for differentiation.

Bees likely rely on floral and/or pollen color (i.e. vision), floral and/or pollen odor (i.e. olfaction) and/or pollen taste (i.e. their sensitivity to chemotactile cues) as cues to distinguish between different types of pollen [14, 21, 27, 28]. Honeybees (*Apis mellifera*) can learn floral patterns, shapes and colors of different plant species and foraging decisions are often based on such visual cues [14, 29, 30]. Honeybees can also discriminate between many different odors [31–33] and thus various floral scents [34, 35], for example field-bean (*Vicia faba*, Fabaceae) and oilseed-rape (*Brassica napus*, Brassicaceae) pollen scent [36]. They may also use taste/gustatory receptors on the distal segment of their antennae, their mouthparts and the tarsi of their forelegs to perceive water, sugars, salt and possibly other nutrients [28, 37]. Because it is still unknown whether taste reception via touch is primarily chemical, tactile or a mix of both, we generally refer to taste or gustatory cues as chemotactile cues. Notably, the use of such chemotactile cues for pollen differentiation has as yet not been studied in honeybees [37]. This is surprising given that resource nutritional quality can only be directly inferred from taste perception, which is a prerequisite for selecting e.g. the currently “best” pollen type directly in the field. Moreover, several studies (reviewed in [14]) indicate that honeybees are, just like bumblebees [27, 28], able to assess pollen nutritional quality.

In this study, we investigated the contribution of the major senses to pollen type differentiation using differential conditioning of the proboscis extension response (PER), a behavior which relies on the bees’ innate response to sugar water and is used in many studies investigating learning and memory formation in (honey)bees (e.g. [38, 39, 40]). We tested whether honeybees can discriminate between two pollen types using (i) only olfactory cues, (ii) olfactory and chemotactile cues and (iii) olfactory, chemotactile and visual cues. Experiments were further conducted in two different seasons (summer and winter) to account for possible effects between these two groups on learning performance. Based on the previous work by Cook et al. [36], we expected that honeybees were able to distinguish between the two different pollen types offered (i.e. almond (*Prunus dulcis*, Rosaceae) and apple (*Malus domestica*, Rosaceae) pollen) based on olfactory cues alone. We further hypothesized that access to both chemotactile and visual cues would improve their differentiation ability, as discrimination is improved by using several interrelated cues [14]. We finally assumed that bees tested in summer would show better performance in pollen differentiation than winter bees, because they are more experienced with the task of differentiating between different pollen types and differ physiologically from winter bees [41].

## Materials and methods

### Study animals and test substances

Experiments were performed with the western honeybee (*Apis mellifera carnica*) at different times of the year. Honeybee colonies were kept at the bee-station at the Biocenter of the University of Würzburg, Germany. In the first and third test period, conducted in August 2016 and May 2018, respectively, leaving forager bees were caught randomly from the entrance of five different hives placed outside in the field (henceforth referred to as summer bees). In the second period, conducted in October and November 2016, honeybees were collected in the same way, but from two hives kept in a heated greenhouse, where they could forage on bee-collected pollen (Naturwaren Niederrhein GmBH, Goch Asperden, Germany), over the winter months (henceforth referred to as winter bees).

Hand-collected apple (*Malus domestica*, Rosaceae) and almond (*Prunus dulcis*, Rosaceae) (anther) pollen (obtained from Firman Pollen, Yakima, WA, USA) were used to investigate the contribution of olfactory, visual and chemotactile cues used for pollen type differentiation. Both pollen types were most likely new to our bees, as both almond and apple pollen was collected from plants grown in the United States. Also, apple flowers in spring. Summer bees in May and August thus hardly encounter apple pollen, unless it was stored for a prolonged period and then also processed and mixed with other pollen. Such pollen most likely strongly differs from the fresh non-processed pollen used in our experiments. Pollen was placed on a wet filter paper to test olfactory cues, and pollen was mixed with de-ionized water (60 ml apple pollen + 55 ml water, 60 ml almond pollen + 60 ml water; different amounts of water were added to reach a similar consistency) to create a paste, which could be applied to the plates for testing chemotactile cues ([27], see below).

The amino acid contents of both pollen types were analyzed using ion exchange chromatography (IEC) (for a detailed method description see [27]).

### Experimental setup

The following restraining procedure was adapted from Bitterman et al. [40]. Upon capture from hives, foragers (between ten and 20 individuals per container) were chilled on ice for about 10 min to reduce their activity. They were then harnessed in plastic tubes (25x10 mm) made from pipette tips and fixed with two crepe tape strips (Hartenstein, Würzburg, Germany). A broad strip (10 mm) was wrapped around the tube horizontally to prevent honeybees from moving their abdomen, while a smaller strip (1 mm) was placed between the bee’s head and thorax and allowed free movement of the antennae, mandibles and proboscis [40]. All restrained individuals were fed 4 μl of 30% w/w sucrose solution with a micropipette and finally kept for 3 h in a climate chamber at 25°C at a relative humidity of 50%.

All experiments were conducted in a temperature constant room (≈ 22°C) at the University of Würzburg, Germany. The experimenter always wore latex gloves to avoid interference of pollen odors and the smell of human skin. After three hours starvation time, each bee was tested for a proper PER by presenting 30% w/w sucrose solution with a toothpick to their antennae. We used only those bees that extended their proboscis upon this gustatory stimulation (about 80% of the bees) for the following experiments, while all other bees were discarded. All individuals were used in one experiment only.

#### Differential PER conditioning

All conditioning experiments were adapted from Ruedenauer et al. [27]. For differential conditioning, we used two conditioned stimuli (pollen types) and an unconditioned stimulus (US: sucrose) as reward. However, in contrast to classical PER conditioning, where the CS is neutral at the beginning and bees usually do not respond upon presentation, almost all honeybees spontaneously extended their proboscis after they received the pollen odor (see below). Consequently, the learning curves in our experiments usually started at high response levels and decreased in the course of the experiment, when the bees had learned that the non-rewarded stimulus was not rewarded with sucrose. We therefore refer to all tested stimuli as S instead of CS, because CS typically defines a neutral stimulus, which, as turned out, was not the case in our learning experiments (see below). These experiments should therefore not be compared to classical PER conditioning, and the percentage of bees showing a PER should not be seen as learning performance, as is usually is the case in classical PER conditioning. Based on our results, we can however make inferences on whether bees can differentiate between the two pollen types.

To test whether bees were able to distinguish the two different pollen types, one pollen type (S+) was rewarded with an unconditioned stimulus (US) (i.e. sucrose solution) (as for CS in [40]). The US was presented with a toothpick covered with 30% w/w sucrose solution, touching one of the bees’ antennae, and the bee was allowed to lick the toothpick. The second pollen type remained unrewarded (S-). If bees were able to discriminate S+ and S-, they should only show a PER when the S+ was presented in anticipation of the associated US. Both pollen types were used as S+ and S-, respectively, with a similar number of bees tested.

For all conditioning experiments, we used a standard protocol established for bees by Bitterman et al. [40]. Each individual went through 20 trials (10 S+ and 10 S- trials) presented in a pseudo-randomized order, with an inter-trial interval (ITI) of 8 min. In the first 15 s of each trial, the individual bee was allowed to rest and habituate to the setup. Then, the S was presented for 6 s. In the case of a rewarded trial, the US was offered in addition to the S+ by briefly touching one antenna with sucrose solution 3s after the S+ presentation started. The bee was then allowed to lick the reward as soon as it extended its proboscis. In an unrewarded trial, only the S- was presented for 6 s. Finally, the trial ended with a period of another 15 s resting time before the bee was replaced by the next individual.

To test whether olfactory cues were sufficient to enable honeybees to distinguish between apple and almond pollen, 10 mg, 50 mg and 300 mg apple or almond pollen were placed on a wet filter paper inside a 20 ml syringe. Different amounts were used to test whether pollen (and thus odor) amount affected learning. Even though equally large pollen amounts are clearly not found at flowers, bees may still encounter large amounts of pollen stored in their nests.

The used filter paper equaled the size of the diameter of the syringe to avoid spillage of pollen. To prevent the plunger from touching the pollen while pressing, a pin was pierced through the syringe at its 4 ml mark. For presentation of the S, the syringe was at maximum filled with air and the plunger pressed slowly downwards until the pin stopped it. The so produced airstream was directed at the bees’ antennae forcing bees to rely on pollen odor only to distinguish between pollen types. Overall, 120 bees were tested during olfactory conditioning (ten bees per experimental round, only summer bees captured in May).

To test for the importance of chemotactile cues, additional experiments were performed using cupreous sticks with a small plate (3x4 mm) at one end [42]. For the S, 50 mg of the pollen pastes were applied to the plate, which was then moved towards one of the bees’ antennae by means of a micromanipulator and touched the antenna for 6 s. After the trial, all plates were cleaned in 70% ethanol [27]. Overall 96 bees were tested in chemotactile conditioning, 32 summer bees and 64 winter bees (eight bees per experimental round).

To test for the importance of visual cues, chemotactile conditioning was performed under both, red light conditions with a spectrum larger than 640 nm (*N* = 32 individuals), which they cannot perceive [43, 44], and daylight conditions (i.e. light from outside plus fluorescent tubes in the laboratory, *N* = 32 individuals) where bees could not only touch, but also see the pollen pastes tested and thus may use visual cues in addition to chemotactile (and olfactory) cues. Here, only winter bees were tested.

#### Unrewarded PER experiment

Our experiments revealed that honeybees did not differentiate between the two different pollen types when only olfactory cues were presented (see below), likely because pollen odor alone evoked a spontaneous PER, similarly to the innate PER shown in response to sugar solution. This spontaneous response had also been observed in previous studies [14, 45]. In contrast, honeybees were able to differentiate between the different pollen types when (additional) chemotactile cues were accessible (see below), likely because they now either suppressed the spontaneous PER in response to the S- or maintained high levels of PER across all trials in response to S+. In order to differentiate between the two possibilities, we performed an additional experiment in April 2017 by repeating the chemotactile conditioning experiment in the dark (to exclude visual cues). Now, half of the tested individuals did not receive a (sugar) reward (but both of the S, i.e. both pollen types) over the entire experiment, while the other half was differentially conditioned as before (see above), with individuals of both groups being tested simultaneously. The order of pollen types (or S+ and S-) was the same as described above. For non-rewarded individuals, we therefore refer to the two different pollen types as pseudo-S+ and pseudo-S-. We assumed that if differentiation in the rewarded experiment was due to suppressing the spontaneous PER, the spontaneous response should be maintained throughout the 10 trials in the unrewarded experiment. Alternatively, if differentiation in the rewarded experiment was caused by keeping the PER response to the S+ high, the spontaneous response should gradually drop in the unrewarded experiment.

### Statistical analysis

All statistical tests were conducted using R v 3.3.2. For olfactory and chemotactile differential conditioning experiments, the number of PER to each S were summed up and used as response variable, ranging between 0 and 10 for each bee (see [27]). For all conditioning experiments, generalized linear mixed effect models (GLMMs) with Poisson distribution were used (*glmmML* package) with bee individuals as random factor to account for repeated testing of the same bee (and thus data dependency). We first tested whether the interaction between stimulus (i.e. S+, S-) and pollen type (apple, almond) significantly affected the number of PER. We found no significant effect for this interaction neither for olfactory (*z*_73_ = -0.554, *P* = 0.579, S1 Fig) nor chemotactile trials (*z*_187_ *=* 1.791, *P* = 0.073, S2 Fig), indicating that the type of pollen used for S+ and S- did not affect the bees’ learning performance [27]. Therefore, only stimulus (S+, S-) was tested for a significant effect on PER numbers in a second set of models.

We additionally tested for a significant effect of the interaction between stimulus and illumination (light, dark) and the interaction between stimulus and season, as chemotactile differential conditioning experiments were further conducted at two different time periods. Both season and illumination significantly interacted with stimulus type in affecting PER numbers (see results). Therefore, separate GLMMs were performed for each group (i.e. summer and winter bees, and bees tested in light and dark) to test for the effect of stimulus on PER numbers in each group. Note that the same group of winter bees (i.e. 32 individuals) tested in chemotactile conditioning in light was included twice in our models (for the summer–winter and light–dark comparison). Because of multiple testing of the same dataset, we finally performed a *P*-value adjustment using Bonferroni correction. All *P*-values remained significant after correction.

To test whether bees tested at different times showed differences in their general response behavior prior to conditioning, we compared the number of spontaneous responses in the first trial across all three experiments using a Chi-squared homogeneity test. We found no significant differences in the first responses between groups (*Chi*^*2*^_2_ = 0.82, *P* = 0.663, S3 Fig), indicating that all study bees shared the same initial response behavior.

To compare the amino acid profiles of both pollen types we also used a Chi-squared homogeneity test.

In the unrewarded PER experiment, we applied generalized linear models (GLM) with Poisson distribution to compare differences between pollen types and (pseudo-) S+ and S- followed by a Tukey test for multiple comparisons.

## Results

### Differential PER conditioning of olfactory cues

**Fig 1:**
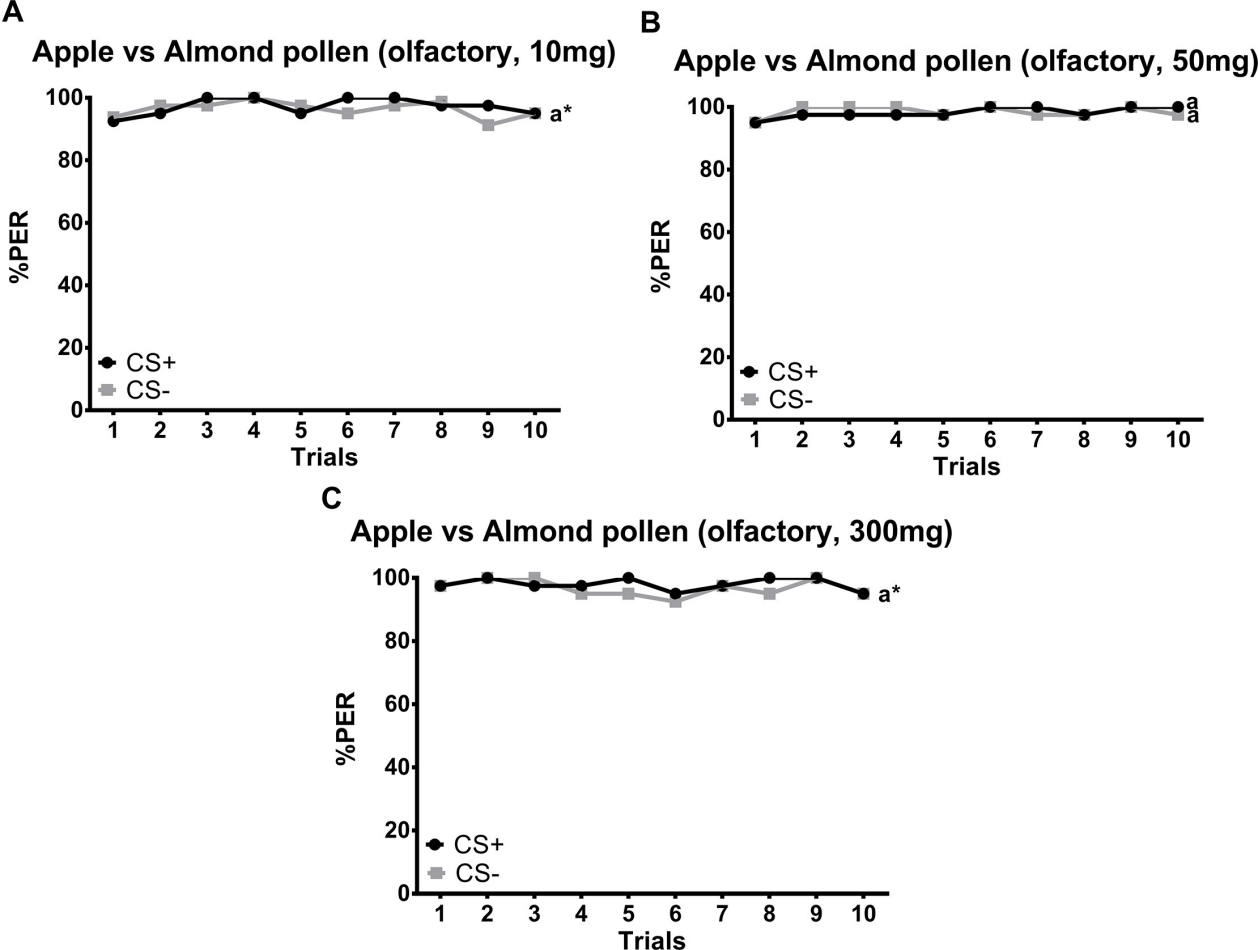
**Percentage of proboscis extension responses (%PER) shown by *Apis mellifera* individuals (*N* = 120) in differential olfactory conditioning to the odor of (A) 10 mg (N = 40), (B) 50 mg (N = 40) and (C) 300 mg (N = 40) of apple versus almond pollen over 10 trials.** S+ (black) represents the rewarded conditioned stimulus, S- (grey) the unrewarded conditioned stimulus. Both, apple and almond pollen were used as S+ and S-. As there was no significant difference in learning performance between apple and almond pollen odor used as S+ or S- (*z*_227_ = 0, *P* = 1, S1 Fig), both groups were summarized into one. Similar letters next to each line indicate no significant difference between stimuli (*P* > 0.05). Asterisks indicate overlapping letters.

Honeybees were not able to distinguish between apple and almond pollen, based on olfactory cues alone (*z*_237_ = 0.145, *P* = 0.885; Fig 1), independent of pollen amount. Interestingly, the majority of all tested individuals (>90%) showed a spontaneous PER to pollen odors immediately after the first presentation of the S (pollen) and thus before the US (sugar solution) was provided. Moreover, the average response rate to both S+ and S- remained high over all ten conditioning trials (i.e. above 85%, Fig 1).

### Differential PER conditioning of chemotactile cues

**Fig 2:**
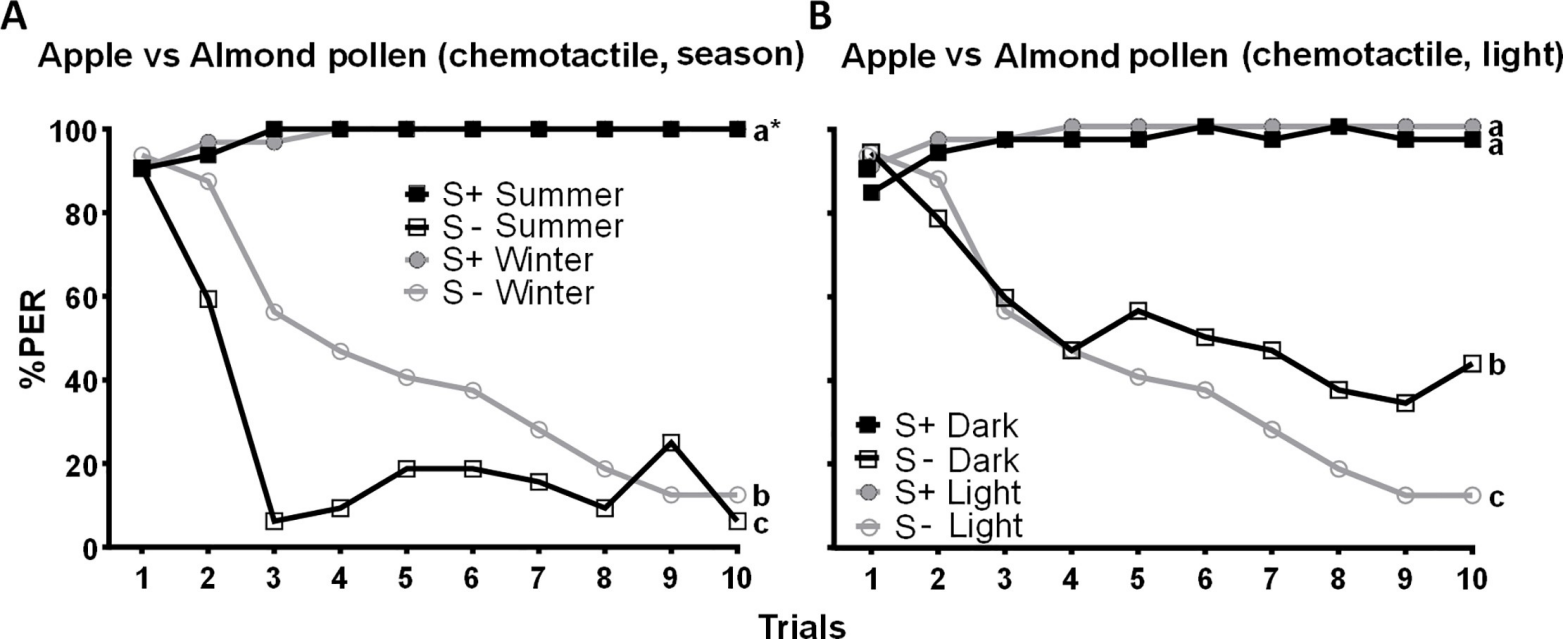
**Percentage of proboscis extension responses (%PER) shown by *Apis mellifera* individuals in differential chemotactile conditioning to the taste of apple versus almond pollen over ten trials tested in (A) different seasons (*N* = 64) and (B) with and without the availability of visual cues (*N* = 64).** S+ represents the rewarded conditioned stimulus, S- the unrewarded conditioned stimulus. Both apple and almond pollen were used as S+ and S-. As there was no significant difference in learning performance between the taste of apple and almond pollen used as S+ or S- (*z*_187_ *=* 1.791, *P* = 0.073, S2 Fig), both groups were summarized into one. (A) Differential conditioning of chemotactile cues in summer (square, *N* = 32) and winter (circle, *N* = 32). (B) Differential conditioning of chemotactile cues in winter in light (circle, *N* = 32) and darkness (square, *N* = 32). Different letters next to the lines indicate significant differences between groups (Tukey test for the models comparing S+ and S-, *P* < 0.001 for all differences). An asterisk indicates the same letter for two overlaying curves.

When honeybees were allowed to touch the pollen paste with their antennae, they were able to distinguish between apple and almond pollen (*z*_189_ = 14.34, *P* < 0.001, Fig 2). Again, a high proportion of individuals showed a spontaneous PER in the very first trial (84–91%), independent of season or setup (S3 Fig), but the number of PER responses towards S- decreased in subsequent trials, unlike those towards the S+ (Fig 2).

Both summer- and winter bees were able to distinguish the two pollen types (summer bees: *z*_61_ = 10.335, *P* < 0.001; winter bees: *z*_125_ = 9.874, *P* < 0.001), but winter bees required more trials to reach the same differentiation level as summer bees (significant interaction: *z*187 = -4.597, *P* < 0.001; Fig 2A, S4 Fig).

Likewise, bees tested in light and in darkness were both able to distinguish the two pollen types (light: *z*_125_ = 13.36, *P* < 0.001; darkness: *z*_61_ = 5.934, *P* < 0.001; Fig 2B), but bees tested in light showed an overall higher learning performance (significant interaction: *z*_187_ = 3.919, *P* < 0.001; Fig 2B).

### Unrewarded PER experiment

**Fig 3:**
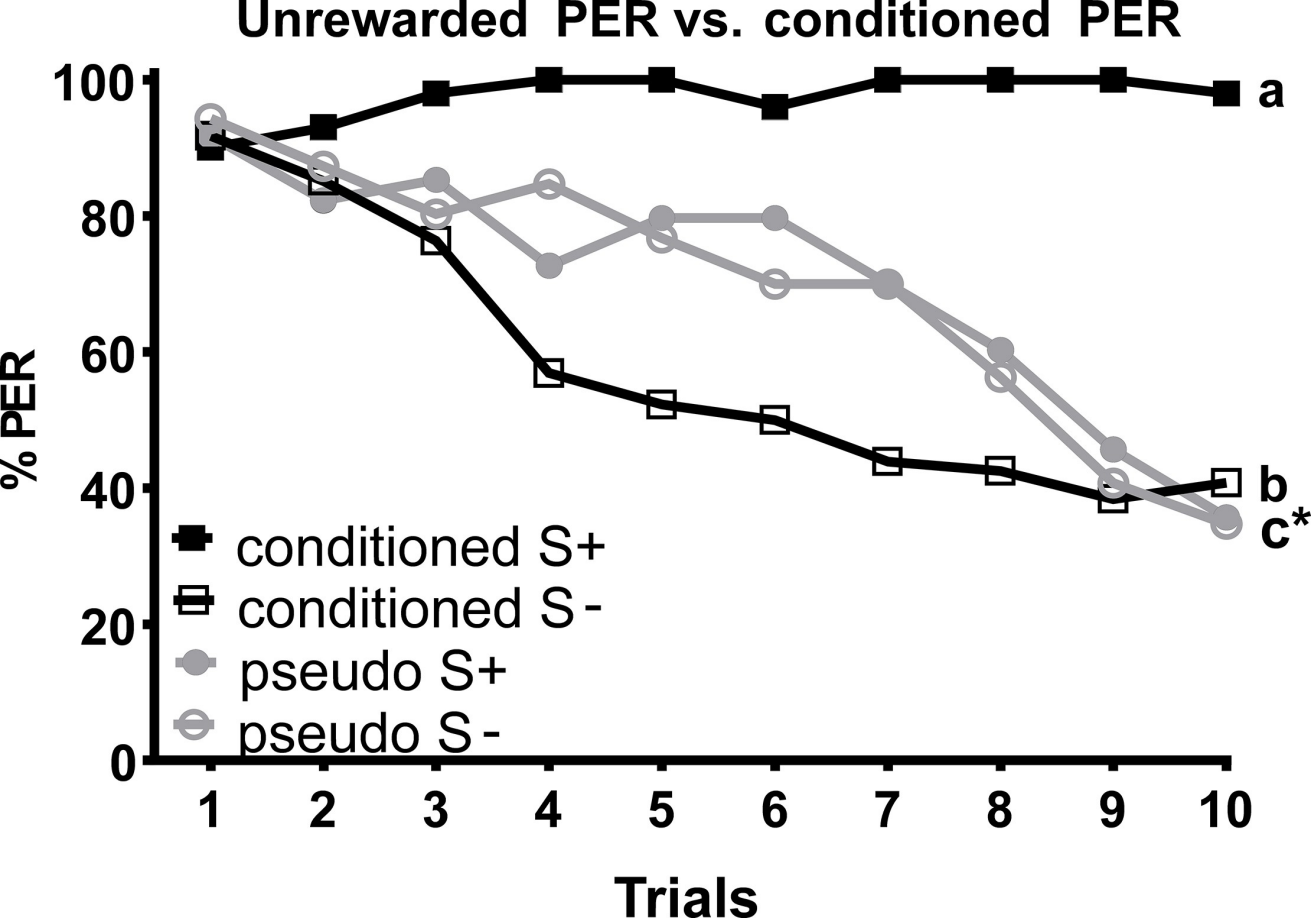
Percentage of proboscis extension responses (%PER) shown by *Apis mellifera* individuals in the unrewarded trials and differential chemotactile conditioning in the dark to the taste of apple versus almond pollen. Bees of the four groups were tested within the same experimental series. As before, the conditioned S+ and S- (*N* = 20) represent the rewarded and unrewarded conditioned stimulus. In contrast, both pseudo-S+ and S- represent unrewarded stimuli presented to a second group of individuals (*N* = 20). Both apple and almond pollen were used as (pseudo) S+ and S-. As there was no significant difference in PER rate between the taste of apple and almond pollen used as (pseudo) S+ or S- (z_87_ = 0.608, *P* = 0.543), both groups were summarized into one. Different letters next to the lines indicate significant differences between groups. An asterisk indicates the same letter for two overlaying curves.

In the unrewarded PER experiment, no difference was found for the PER rates towards the two different pollen types (*z*_89_ = -0.753, *P* = 0.451). However, responses to the four stimulus types (i.e. S+, S-, pseudo-S+ and pseudo-S-) differed significantly (*z*_89_ = 2.874, *P* = 0.027; Fig 3). The two pseudo-S rates were similar, but differed from both conditioned S, while the differences between rewarded S+ and S- remained significant (Fig 3).

## Discussion

The ability to discriminate different pollen types would be highly beneficial for bees for optimizing their nutritional intake [4, 21, 27]. Contrary to our hypothesis and unlike bumblebees [27], honeybees were not able to distinguish between apple and almond pollen based on olfactory cues alone, but needed (additional) chemotactile cues. However, the bees clearly perceived the presented pollen scents as demonstrated by the high rate of spontaneous PER for both pollen odors at the beginning of the conditioning experiments (above 90%; Fig 1).

The differences between our findings and the results of Cook et al. [36] may be explained by the different pollen types tested. While Cook et al. [36] selected pollen from different plant families (Fabaceae and Brassicaceae), we used apple and almond pollen, which both belong to the Rosaceae. We can thus not rule out family-specific similarities in odor composition, which may have rendered distinction between the odor of apple and almond pollen more difficult for honeybees [46–49]. In fact, bees can discriminate similar odors worse than dissimilar odors [50], but can improve discrimination upon repeated exposure [50–52]. Alternatively (or additionally) different results may be explained by different experimental setups. While Cook et al. [36] used glass wool (which may filter out components that elicit a spontaneous PER), we placed pollen on a humidified filter paper, which may have dissolved additional pollen odor compounds and thereby have provided different cues eliciting spontaneous responses. Cook et al. [36] further used bee-collected pollen in their differential experiments, which could contain additional volatile substances not found in pure pollen as used in our study. The pollen used by Cook et al. [36] may however be comparable to pollen stored in hives and their bees’ responses thus represent in-hive situations, whereas our results rather represent foraging decisions in the field.

It cannot be entirely ruled out that honeybees were able to distinguish between the odor of apple and almond pollen but this was masked by the high rate of spontaneous PER. In fact, olfactory cues alone may not have been sufficient for suppressing their spontaneous proboscis extension response. This may be one reason why honeybees showed similarly high response rates for S+ and S-, whereas bumblebees (which did not respond spontaneously) were able to distinguish almond from apple pollen by odor cues alone [27]. One explanation for why bumblebees did not respond spontaneously may be their overall lower motivation to extend their proboscis [53]. Alternatively, bumblebees may rely on different components of the presented pollen odors for decision-making. Moreover, bumblebees assessed pollen quality and selected pollen of higher quality in a choice experiment [22, 27], whereas comparable studies on honeybees did not find any preferences for high-quality pollen [21, 54]. Unlike honeybees with their mass recruiting dance language, individual bumblebee foragers also tended to rely more on “personal information” than on “social information” [55] and do receive little feedback from larvae or nest-mates [56]. In turn, individual (recruited) honeybees may not themselves assess pollen quality, but rather rely on feedback from nest-mates [21], reducing the need for nutrient selective foraging.

Contrary to the olfactory conditioning experiment where honeybees showed similar response rates to S+ and S- (Fig 1), they clearly differentiated between apple and almond pollen in chemotactile experiments (Fig 2), indicating that honeybees were able to suppress their (spontaneous) proboscis extension reaction to the S- when chemotactile cues were available in addition to olfactory cues. Thus, chemotactile cues appear to enable honeybees to overcome their spontaneous PER response and to build an association between the S+ and the reward. In fact, the differences between pseudo-S and conditioned S- in the unrewarded PER experiment suggest that conditioned bees suppress their spontaneous PER at least to some extent. Additionally, after ten trials, both pseudo-S as well as the conditioned S- resulted in a lower response level (about 40%) than the S+ (>90%), further indicating that the PER to the conditioned S+ is maintained by the reward. Consequently, differentiation in the chemotactile conditioning experiments was due to both suppressing the PER to the S- and maintaining the PER to the S+.

We suggest that the ability of honeybees to differentiate between the two pollen types was largely based on perceiving differences in the nutritional profile of the two pollen types. Protein (i.e. amino acids), fat (i.e. fatty acids) and sugars are the most common nutrients in pollen [3]. Moreover, the amino acid profiles of the two pollen types significantly differ (see S1 Table), rendering amino acids suitable cues for discrimination. However, other non-volatile cues, e.g. sugars, fatty acids and secondary substances, such as flavonoids, may also serve as alternative or additional cues.

Moreover, when we prevented honeybees from perceiving visual cues and thus from using the visual differences between (red-orange) apple and (yellowish) almond pollen in chemotactile experiments, they were still able to differentiate the two pollen types, but learning performance was significantly lower (i.e. 44% of bees tested in darkness still responded to the S- by the end of ten trials compared to only 13% of bees tested in light), which would provide further evidence for the importance of visual information in supporting differential learning and thus foraging decisions in bees [29, 57–59]. Alternatively, the bees tested in light and darkness may have differed in their internal state and therefore motivation in their sensory sensitivity towards the stimuli. However, because response rates in the first trials of our experiments did not differ between winter bees tested in light and bees tested in darkness (S3 Fig), we consider light induced differences in motivation or sensory sensitivity rather unlikely.

We thus suggest that the combination of several (i.e. olfactory, chemotactile and visual) cues, a situation which more closely resembles natural conditions, likely facilitates successful distinction between different rewards. The combination of sensory cues provided by a potential resource (e.g. pollen) likely conveys important information used by bees to assess its properties, which can then be used to learn differences between different resources (e.g. pollen types) and thus to make foraging decisions based on different resource qualities. However, multimodal cues can also reduce the ability of the bees to make the correct choices [60].

With regard to seasonal effects, winter bees needed more trials to reach a similar learning performance than summer bees (conditioned in August, S4 Fig), which agrees with our expectation and with Scheiner et al. [61], who also observed overall higher learning performances in bees tested in August. This finding may be explained by the summer bees’ experience in foraging on flowers in the field, whereas the winter bees were confined to a strongly impoverished foraging environment in the glass house. In fact, summer and winter bees kept under constant conditions in the laboratory without access to floral resources showed no difference in learning performance [62]. Differences in learning performances may further be influenced by differences in juvenile hormone titers [63] known to affect learning [64, 65], associated inactivity and entailed changes in the organization of mushroom bodies [61, 63, 66]. However, we cannot rule out that the differences found resulted from physiological characteristics specific for the 2016 winter and summer cohort. As we found no differences when comparing the number of bees spontaneously responding to the stimulus in the first trial between cohorts, we can at least presume that the summer and winter cohorts shared the same response behavior prior to conditioning (see S3 Fig).

Also note that collected pollen can be stored in the nest over prolonged periods, which can alter its chemical composition following microbial processing [67, 68] or enrichment with bee salivary compounds [69]. How such chemical modification affects interactions between bees and pollen is largely unknown.

In summary, we conclude that honeybees rely on several sensory cues (i.e. olfactory, chemotactile and visual stimuli) to most effectively differentiate between different pollen types, which likely represents the most natural condition, as foraging resources typically provide more than one sensory cue. Under natural conditions, individual honeybee foragers are therefore able to differentiate different pollen types and potentially select those pollen types, which best support an optimal diet.

## Supporting information

S1 Fig Percentage of proboscis extension responses (%PER) shown by *Apis mellifera* individuals (*N* = 39) in differential olfactory conditioning to the odor of apple versus almond pollen over 10 trials with separate lines for rewarded (S+, filled symbols) and unrewarded (S-, clear symbols) stimuli. Both, apple (grey) and almond (black) pollen were used as S+ and S-. There was no significant difference in learning performance between apple and almond pollen odor used as S+ or S- (*z*_73_ = -0.554, *P* = 0.579).
(TIF)

S2 Fig Percentage of proboscis extension responses (%PER) shown by *Apis mellifera* individuals (*N* = 64) in differential chemotactile conditioning in the dark to the taste of apple versus almond pollen over 10 trials with all stimuli separated. S+ (filled) represents the rewarded conditioned stimulus, S- (clear) the unrewarded conditioned stimulus. Both, apple (grey) and almond (black) pollen were used as S+ and S-. There was no significant difference in learning performance between apple and almond pollen odor used as S+ or S- (*z*_187_ *=* 1.791, *P* = 0.073).
(TIF)

S3 Fig Number of individuals showing a proboscis extension response (PER) (dark grey) and not showing a PER (light grey) in the first trial of all experiments performed. There were no significant differences (n.s.) between different seasons or light conditions (*Chi*^*2*^_2_ = 0.82, *P* = 0.663).
(TIF)

S4 Fig **Number of proboscis extension responses (PER) shown by *Apis mellifera* individuals (*N* = 132) in differential chemotactile conditioning of summer (N = 64, left) and winter (N = 64, right) bees to the taste of apple versus almond pollen. Boxplots display responses to S+ and S-.** S+ represents the rewarded stimulus, S- the unrewarded stimulus. Both, apple and almond pollen were used as S+ and S-. While there was no difference between the S+ between summer and winter bees (GLMM: *z*_93_ *=* -0.185, *P* = 0.853), summer bees responded significantly less to the S- (GLMM: *z*_93_ *=* 4.969, *P* < 0.001).
(TIF)

S1 Table Amino acid content (in μmol/g dry weight) of apple and almond pollen used in the PER experiments: determined via ion exchange chromatography (see [27]). In addition to concentrations of 20 protein-coding amino acids, concentrations for gamma-Aminobutyric acid (GABA) and hydroxyproline are provided.
(DOCX)
