# Supplementary figures and images for: Do honeybees (Apis mellifera) differentiate between different pollen types?
Source: PLoS One. 2018 Nov 7;13(11):e0205821. doi: 10.1371/journal.pone.0205821 (PMC6221266; doi:10.1371/journal.pone.0205821)

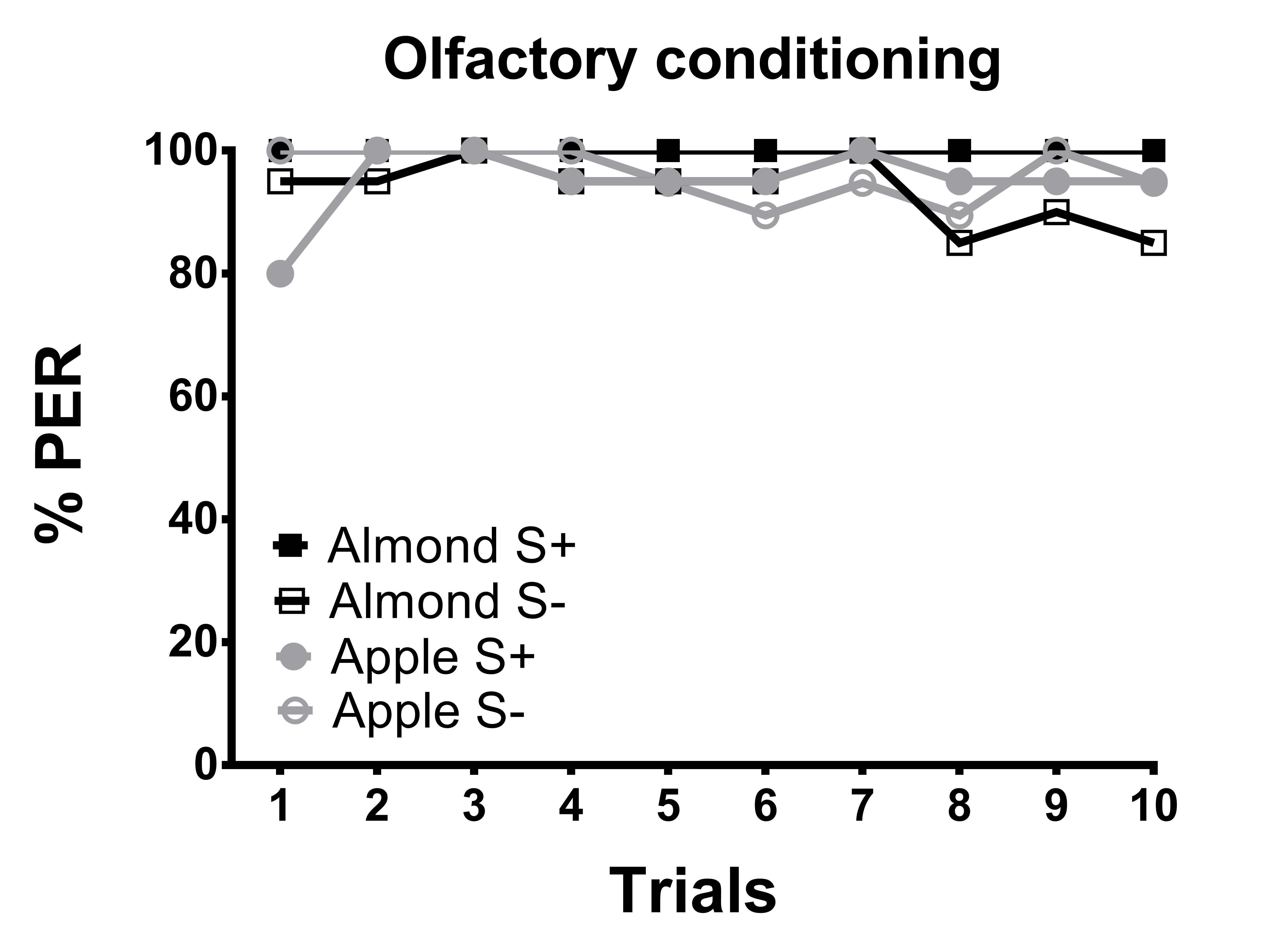

Supplement: S1 Fig — Both, apple (grey) and almond (black) pollen were used as S+ and S-. There was no significant difference in learning performance between apple and almond pollen odor used as S+ or S- (z73 = -0.554, P = 0.579). (TIF) [file pone.0205821.s001.tif]

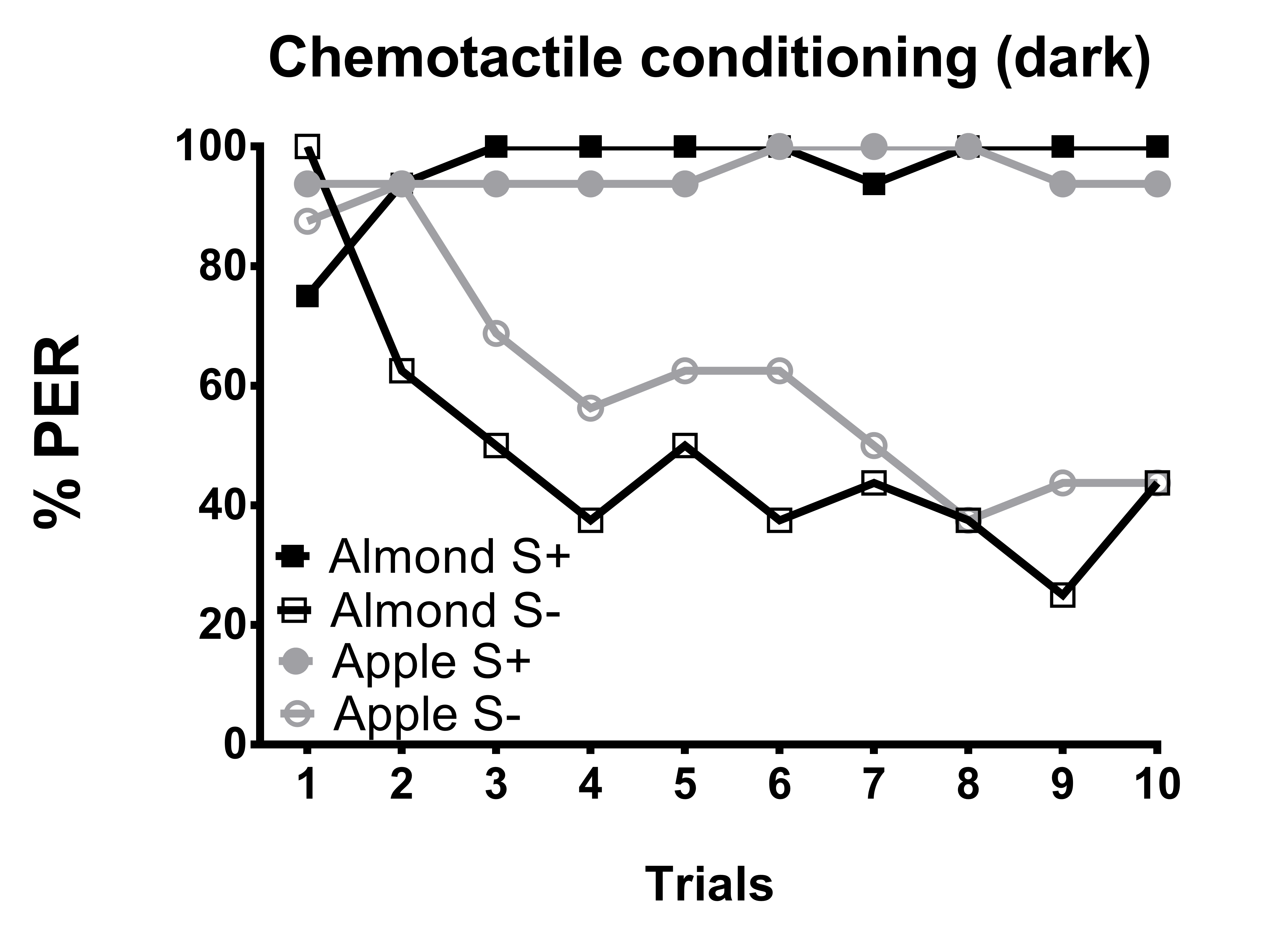

Supplement: S2 Fig — S+ (filled) represents the rewarded conditioned stimulus, S- (clear) the unrewarded conditioned stimulus. Both, apple (grey) and almond (black) pollen were used as S+ and S-. There was no significant difference in learning performance between apple and almond pollen odor used as S+ or S- (z187 = 1.791, P = 0.073). (TIF) [file pone.0205821.s002.tif]

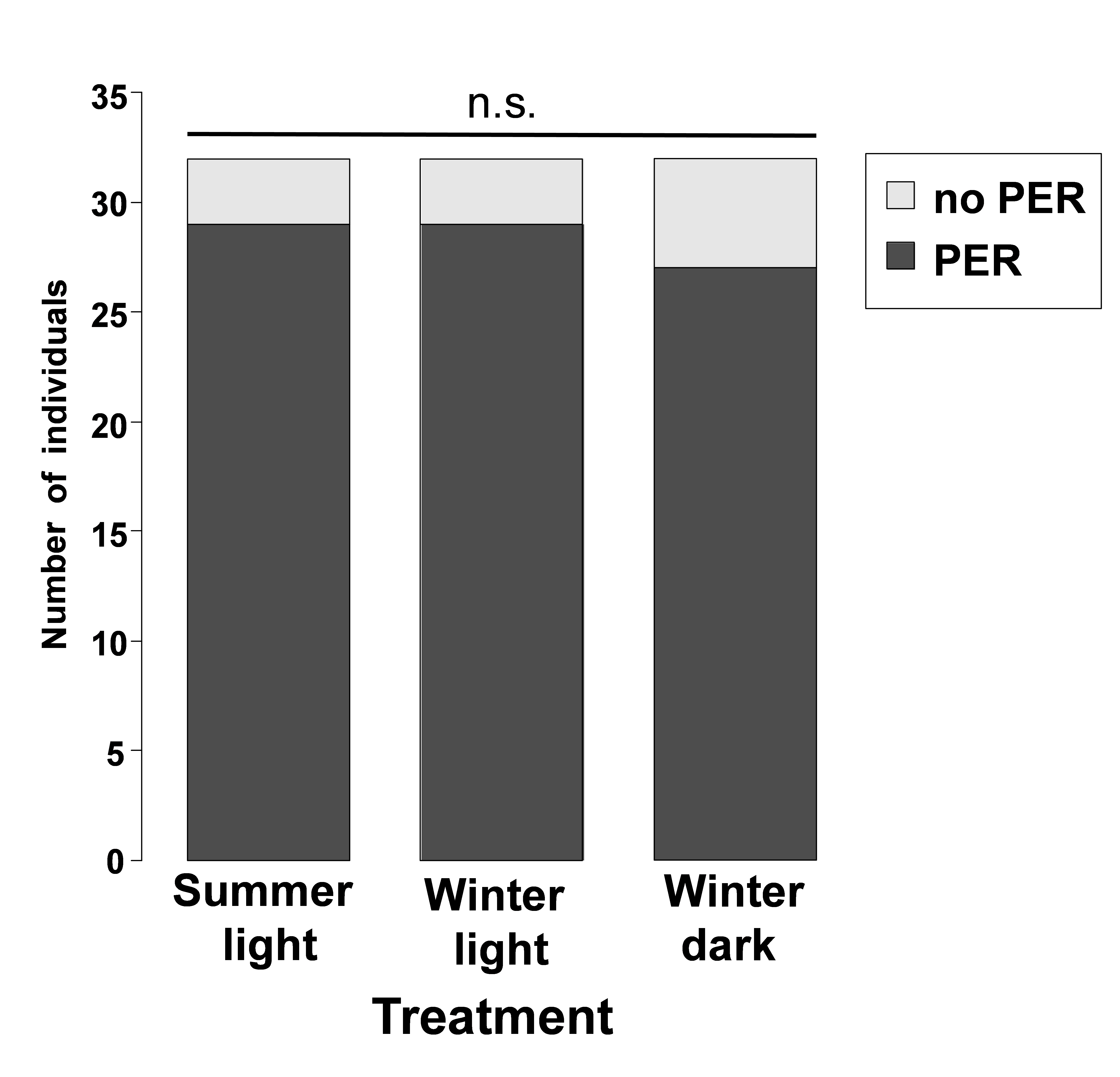

Supplement: S3 Fig — There were no significant differences (n.s.) between different seasons or light conditions (Chi22 = 0.82, P = 0.663). (TIF) [file pone.0205821.s003.tif]

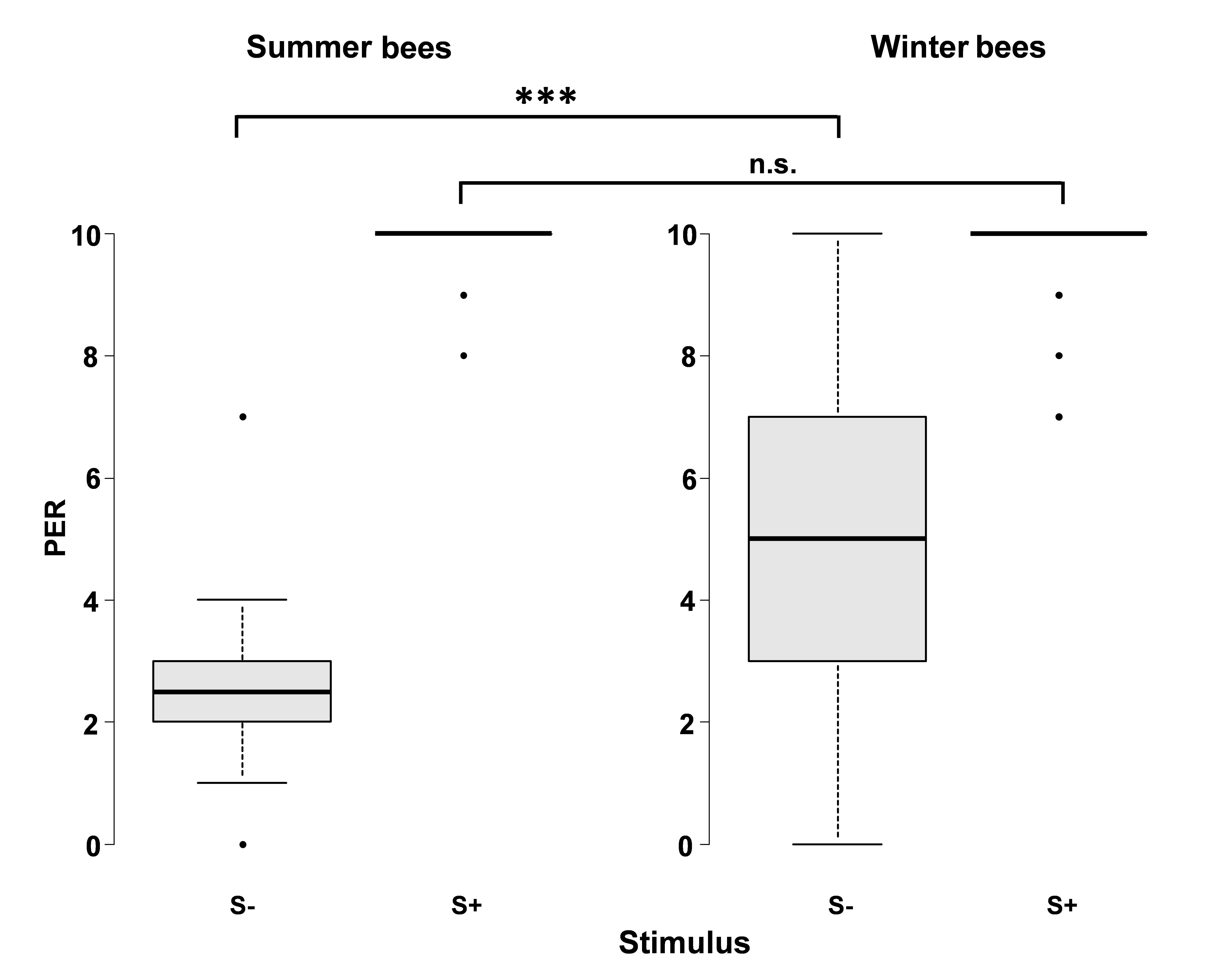

Supplement: S4 Fig — Number of proboscis extension responses (PER) shown by Apis mellifera individuals (N = 132) in differential chemotactile conditioning of summer (N = 64, left) and winter (N = 64, right) bees to the taste of apple versus almond pollen. Boxplots display responses to S+ and S-. S+ represents the rewarded stimulus, S- the unrewarded stimulus. Both, apple and almond pollen were used as S+ and S-. While there was no difference between the S+ between summer and winter bees (GLMM: z93 = -0.185, P = 0.853), summer bees responded significantly less to the S- (GLMM: z93 = 4.969, P < 0.001). (TIF) [file pone.0205821.s004.tif]
