# Supplementary material for: Do honeybees (Apis mellifera) differentiate between different pollen types?
Source: PLoS One. 2018 Nov 7;13(11):e0205821. doi: 10.1371/journal.pone.0205821 (PMC6221266; doi:10.1371/journal.pone.0205821)
Supplement: S1 Table — In addition to concentrations of 20 protein-coding amino acids, concentrations for gamma-Aminobutyric acid (GABA) and hydroxyproline are provided. (DOCX) [file pone.0205821.s005.docx]

**S1 Table. Amino acid content (in µmol/g dry weight) of apple and almond pollen used in the PER experiments:** determined via ion exchange chromatography (see [27]). In addition to concentrations of 20 protein-coding amino acids, concentrations for gamma-Aminobutyric acid (GABA) and hydroxyproline are provided. The amino acid contents of both pollen types differ significantly (chi squared test: *Chi^2^_17_* = 83.379, *P* > 0.001).

|  | **Apple** | **Almond** |
| --- | --- | --- |
| **Amino acid** | **µmol/g** | **µmol/g** |
| Asparagine | 275.80 | 116.32 |
| Hydroxyproline | 1.69 | 6.18 |
| Threonine | 23.29 | 26.12 |
| Serine | 45.73 | 48.96 |
| Asparagine | 0.00 | 0.00 |
| Glutamic acid | 67.19 | 57.59 |
| Glutamine | 0.00 | 0.00 |
| Proline | 77.93 | 131.78 |
| Glycine | 53.04 | 64.36 |
| Alanine | 66.11 | 64.39 |
| Valine | 18.04 | 18.25 |
| Cysteine | 0.00 | 0.00 |
| Methionine | 6.59 | 7.34 |
| Isoleucine | 13.14 | 12.37 |
| Leucine | 36.26 | 40.74 |
| Tyrosine | 8.24 | 9.45 |
| Phenylalanine | 20.86 | 30.67 |
| GABA | 12.99 | 8.43 |
| Lysine | 36.94 | 42.07 |
| Histidine | 9.15 | 9.64 |
| Tryptophane | 0.00 | 0.00 |
| Arginine | 15.29 | 17.02 |
